# Direct Fusion of Geostationary Meteorological Satellite Visible and Infrared Images Based on Thermal Physical Properties

**DOI:** 10.3390/s150100703

**Published:** 2015-01-05

**Authors:** Lei Han, Buzha Wulie, Yiling Yang, Hongqing Wang

**Affiliations:** 1 School of Information Science and Engineering, Ocean University of China, 238 Songling Road, Qingdao 266100, China; E-Mails: hanlei@ouc.edu.cn (L.H.); wuliebuzha@outlook.com (B.W.); 2 State Key Laboratory of Severe Weather, Chinese Academy of Meteorological Sciences, Beijing 100081, China; 3 Department of Atmospheric and Oceanic Science, Peking University, Beijing 100871, China

**Keywords:** geostationary satellite, fusion, infrared image, visible image

## Abstract

This study investigated a novel method of fusing visible (VIS) and infrared (IR) images with the major objective of obtaining higher-resolution IR images. Most existing image fusion methods focus only on visual performance and many fail to consider the thermal physical properties of the IR images, leading to spectral distortion in the fused image. In this study, we use the IR thermal physical property to correct the VIS image directly. Specifically, the Stefan-Boltzmann Law is used as a strong constraint to modulate the VIS image, such that the fused result shows a similar level of regional thermal energy as the original IR image, while preserving the high-resolution structural features from the VIS image. This method is an improvement over our previous study, which required VIS-IR multi-wavelet fusion before the same correction method was applied. The results of experiments show that applying this correction to the VIS image directly without multi-resolution analysis (MRA) processing achieves similar results, but is considerably more computationally efficient, thereby providing a new perspective on VIS and IR image fusion.

## 1. Introduction

Geostationary meteorological satellites often collect data for both infrared (IR) and visible (VIS) channels. The IR data of geostationary meteorological satellites are of great importance in research and practical applications. They reflect the distribution of temperatures on the Earth's surface, and are used widely in weather forecasting, numerical weather prediction, and climate modeling. However, the infrared spatial resolution is relatively low. By contrast, the VIS data have considerably higher resolution, but do not reflect the thermal dynamics of the Earth and atmosphere. The fusion of IR and VIS data into one coherent image provides a method of improving the infrared spatial resolution.

Many image processing techniques have been developed to address multispectral data fusion. Common multi-resolution analysis (MRA) techniques, such as the Laplacian pyramid [1], wavelet [2–4], curvelet [5,6], and contourlet [7,8] are capable of enhancing the visual resolution [9], but fail to incorporate the underlying physical properties. The spectral identity of the fused result is not clearly defined and the images often suffer from spectral distortion. Other methods are aimed directly at fusing multisensor satellite images, such as the Brovey method [10], pixel block intensity modulation (PBIM) [11], smoothing filter-based intensity modulation (SFIM) [12], the Choi method [13] and Aanaes method [14]. However, these approaches cannot be applied directly to the fusion of VIS and IR images fusion from geostationary meteorological satellites.

Previously, we proposed a *post hoc* physical correction method for VIS and IR data fusion to obtain higher-resolution IR images [15]. This method [hereafter Han2014] comprises two steps. In the first step, the high-resolution structural features of the VIS image are extracted and incorporated into the IR image using a regular multi-resolution fusion approach, such as multiwavelet analysis. This step significantly increases the visual details in the IR image, but fake thermal information might be included. In the second step, the Stefan-Boltzmann Law is applied to correct the distortion, to retain or recover the thermal infrared nature of the fused image. The final fused image has higher spatial resolution and retains the fidelity of the original IR thermal information.

In Han2014 [15], the preservation of the infrared physical properties depends heavily on the second-step, *i.e.*, post-physical correction. Then, an interesting question arises: is multi-resolution image fusion necessary in the first place, or can this physical correction be applied to the VIS image directly without any MRA processing? In this study, we attempt to answer this question. The same physical correction algorithm was used, but its input was changed to the VIS image, instead of the synthesized VIS-IR image produced by the multiwavelet fusion. If this approach were effective, it would be a considerably simpler and faster alternative to the previously proposed two-step method, and would provide a new method of fusing VIS and IR images.

## 2. Method

Satellite infrared sensors detect the thermal radiation emitted from the surface of the earth. This raw radiation energy is converted into brightness temperatures, which are then readily available for various applications. This process can be reversed and the brightness temperatures can be converted back into thermal radiation energy. Therefore, to ensure the infrared thermal property in the fused image, the fused image should exhibit a similar level of infrared radiation energy as the original IR image. Based on this, we use the thermal radiation energy calculated from the original IR image as a strong constraint during correction of the fused image.

In Han2014 [15], this physical correction process was performed on the VIS-IR composite image. In this exploratory study, we were interested in the feasibility of correcting the VIS image directly. The VIS image *per se* is considered the pre-correction image, with considerable spectral distortion. The ultimate objective is to obtain higher-resolution IR images without significant spectral distortion.

As in the previous study, all physical correction is performed at the level of the infrared thermal radiation map. We first convert both the IR and VIS images into their corresponding thermal radiation maps based on the Stefan-Boltzmann Law:

(1) $$j=\varepsilon \sigma T^{4}$$

where *j* represents the total energy radiated per unit surface area per unit time, *T* is the temperature in Kelvin, *ε* is the emissivity, and *σ* denotes the Stefan-Boltzmann constant. Let *j_IR_* and *j_VIS_* denote the radiation maps corresponding to the original IR and VIS images respectively. We assume *ε* = 1 to simplify the modeling.

**Figure 1.**
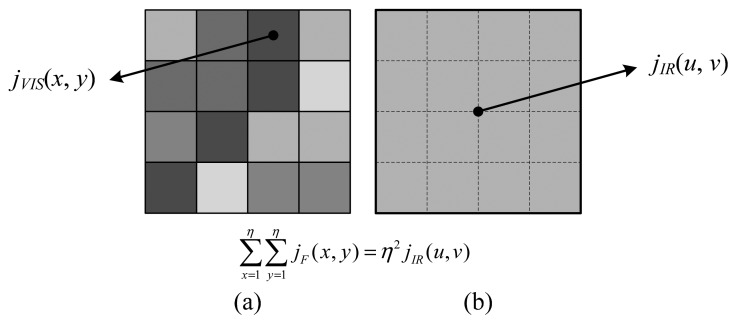
Illustration of physical correction. (**a**) An *η* × *η* window in the VIS image. Here, *η* = 4; (**b**) One pixel in the low-resolution IR image. It is interpolated to the same scale as (a). Both windows in (a) and (b) should have an identical amount of radiation energy.

Then, we traverse the two radiation maps to adjust the regional radiation energy. This is the key step which preserves the thermal physical properties of the final fused image. Specifically, we scale the radiation in each window in the VIS image, *j_VIS_*, to equalize it with *j_IR_* in the corresponding region of the IR image. The window size *η* depends on the resolution disparity between the IR and VIS images, so that one single pixel in the IR image corresponds to an *η* × *η* window in the VIS image (Figure 1).

In our case, *η* = 4 because the data have a VIS resolution of 1 km and IR resolution of 4 km. Let *j_F_*(*x, y*) be the adjusted radiation energy at pixel (*x, y*), we expect the following relation holds within each *η* × *η* window:

(2) $$\sum_{x=1}^{\eta }\sum_{y=1}^{\eta }j_{F}\left(x,y\right)=\eta^{2}j_{IR}\left(u,v\right)$$

Accordingly, the old radiation value at pixel (*x*, *y*) in the VIS image should be updated to:

(3) $$\begin{array}{cc} j_{F}\left(x,y\right)=j_{\text{VIS}}\left(x,y\right)\cdot \frac{\eta^{2}j_{IR}\left(u,v\right)}{\overset{\eta }{\underset{x=1}{\text{∑}}}\overset{\eta }{\underset{y=1}{\text{∑}}}j_{\text{VIS}}\left(x,y\right)}, & 1\leq x,y\leq \eta \end{array}$$

In summary, the basic idea of this method is similar to the second step of Han2014 [15]. The major methodological shift from Han2014 is the elimination of the initial multi-wavelet fusion and the VIS image is used directly as the input of the physical correction algorithm.

## 3. Experimental Results and Analysis

The proposed algorithm was tested using data obtained by the Multifunctional Transport Satellite (MTSAT) which is operated by the Japan Meteorological Agency (JMA). We used its VIS (0.55–0.9 μm) and IR1 (10.3–11.3 μm) channels in our experiments. We first analyzed the qualitative effect of the algorithm before using objective parameters to assess its quantitative performance, presented in the next section. Selected results from our previous study were included to compare performance.

### 3.1. Qualitative Analysis

**Figure 2.**
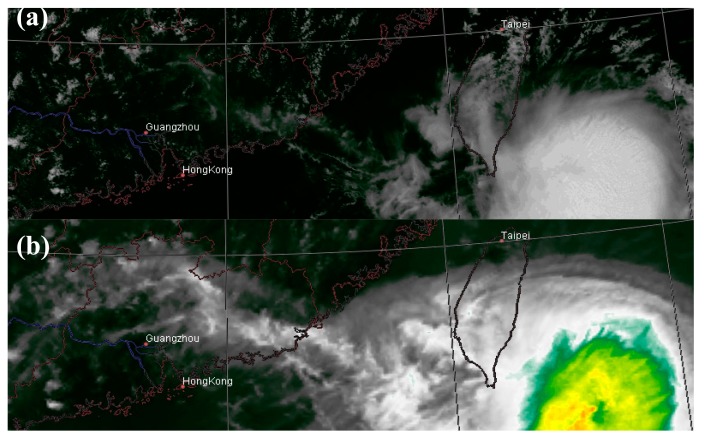

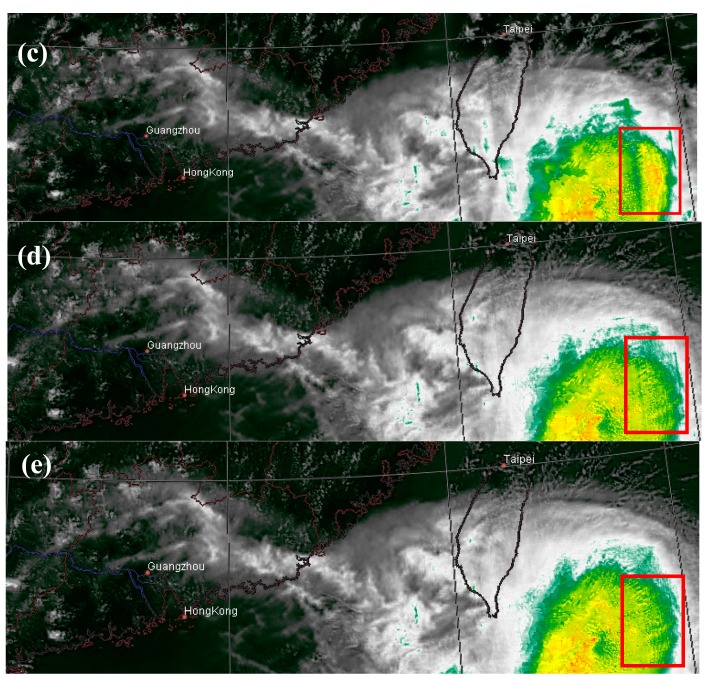
MTSAT images at 1300 LST on 24 July 2006. (**a**) The VIS image fails to capture the peripheral clouds of the cyclone; (**b**) The IR image shows the peripheral clouds that are absent in (a); (**c**) The VIS-IR composite image produced by multiwavelet fusion has higher resolution, and retains the cyclone peripheral clouds as in (b). The red box indicates an area with abnormal temperature; (**d**) Corrected image based on the VIS-IR composite (the final result of Han2014); (**e**) Corrected image based on VIS only (the final result of the new method).

Figure 2 shows a cyclone on 1300 LST 24 July 2006. Figure 2a shows that the VIS channel fails to capture some peripheral clouds of the cyclone which is clear in the IR1 channel in Figure 2b. Figure 2c is the synthesized image based on multiwavelet fusion of the VIS and IR images [15]. Without the physical level correction, the spectral features of the composite image are much distorted. For example, the red box indicates a prominent low-temperature region that does not reflect the actual spectral character. Figure 2d,e shows that both Han2014 and the new method proposed in this study are able to eliminate this distorted temperature. Both methods result in visually similar outcomes and achieve higher resolution than the original IR image.

**Figure 3.**
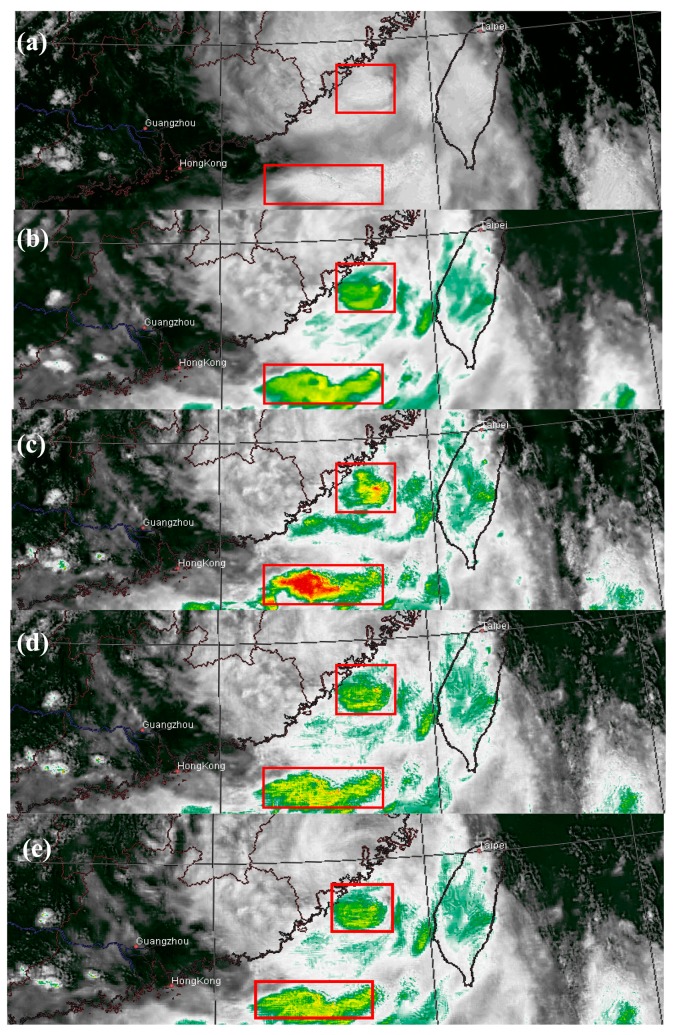
MTSAT images at 1300 LST on 25 July 2006. (**a**) The VIS image; (**b**) The IR image; (**c**) The VIS-IR composite produced by multiwavelet fusion. Red boxes indicate areas with serious spectral distortion; (**d**) The final result of Han2014; (**e**) The final result of the new method.

Figure 3 shows the same region on 1300 LST 25 July 2006. The red boxes indicate where spectral distortion happens. The red boxes in the VIS image (Figure 3a) show two overshooting cloud tops which are very bright. If these pixels are merged using the multiwavelet method, unusually low temperatures will be induced (Figure 3c). Both Han2014 (Figure 3d) and the new method (Figure 3e) are able to correct these abnormally low temperatures to the normal range.

**Figure 4.**
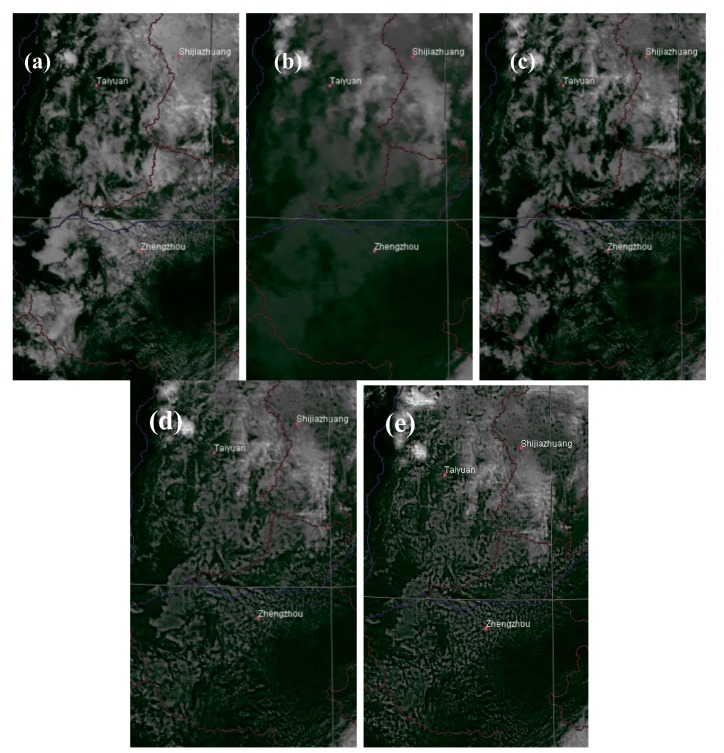
MTSAT images at 1300 LST on 25 July 2006. (**a**) The VIS image; (**b**) The IR image; (**c**) The VIS-IR composite; (**d**) The final result of Han2014; (**e**) The final result of the new method.

Figure 4 shows another area taken on 1300 LST 25 July 2006. Figure 4a shows the VIS image mainly covered by scattered clouds, and the IR image (Figure 4b) shows a relatively high brightness temperature. The multiwavelet fusion of the VIS and IR images yields better visual effects, but the overall temperature in the region is lowered significantly (Figure 4c). The final results of Han2014 (Figure 4d) and the new method (Figure 4e) show that the temperatures in this region are refined to the normal infrared level, while the general cloud pattern from the VIS image is retained.

### 3.2. Quantitative Analyses

We used the same five parameter categories taken from [15] to compare the performance of different methods quantitatively:

(1) Information Entropy (IE) and Mutual Information (MI). IE quantifies the amount of information contained within an image, and MI measures how much information is shared between images. These parameters can characterize the flow of information during the fusion process and the similarity of the synthesized and source images.
(2) Average Gradient (AG). The gradient at a pixel measures how sharply the pixel values change in the surrounding region. Fine details, sharp edges and complex textures produce high varying regional characteristics and are reflected by high gradients. The AG is the average of all regional gradients and reflects the overall sharpness of the image.
(3) Objective Fusion Performance Measure (Qabf). Proposed by Xydeas and Petrović [16], Qabf is a measurement of how much detailed edge information is transferred from the source images to the fused image. This parameter ranges between 0 and 1, where the value 0 represents the complete loss of edge information and 1 represents the perfect preservation of edges.
(4) Universal Image Quality Index (QI) and Edge-dependent Quality Index (QE). QI was initially proposed as a universal measure of image quality by modeling the structural distortion [17], but here we use it as an index of image similarity. This index does not depend on individual observers or testing conditions, and exhibits consistency with subjective evaluations. The dynamic range of QI is [−1, 1] [17]. The closer QI is to 1, the more similar the two images are in comparison.
  QE is adapted from QI such that edge information is taken into account [18]. In addition to the QI of the original images, QE also incorporates the QI of the corresponding edge images obtained from source images. Similarly, the values of QE still range between −1 and 1, with the best value of 1 achieved by perfect image similarity.
(5) Thermal Energy Deviation (AVGD and RMSD). Because the fusion method in this paper is based on thermal radiation, we introduce two new parameters: the average thermal energy deviation (AVGD), and the root-mean-square thermal energy deviation (RMSD). These allow us to evaluate the deviation of thermal energy between the synthesized image and the original IR image.
  If the VIS-to-IR resolution ratio is *η*, a single pixel at (*u*, *v*) in the IR image corresponds to a *η* × *η* area in the fused image. In each *η* × *η* window, the local thermal energy deviation from the original IR image is:
(4) $$\Delta j\left(u,v\right)=\sum_{x=1}^{\eta }\sum_{y=1}^{\eta }j_{F}\left(x,y\right)-\eta^{2}j_{IR}\left(u,v\right)$$
  Then we have the AVGD over the entire image:
(5) $$j_{\text{AVGD}}=\frac{1}{N^{2}}\sum_{u=1}^{N}\sum_{v=1}^{N}\left|\Delta j\left(u,v\right)\right|$$
  Similarly, the RMSD is defined as:
(6) $$j_{\text{RMSD}}=\sqrt{\frac{1}{N^{2}}\sum_{u=1}^{N}\sum_{v=1}^{N}\Delta j{\left(u,v\right)}^{2}}$$

As stated in our previous study [15], using these image-level quality measurements to assess the underlying physical property is a compromise, because few metrics in the literature measure the required physical properties. Fortunately, as shown in the case studies above, the fundamental physical level similarity often externalizes at the image level, so these image-level metrics might be useful to an extent. We also customized two metrics, AVGD and RMSD, to compare the thermal properties of images directly.

Tables 1 and 2 show the results on 1300 LST 24 July 2006. FUS, COR1 and COR2 all have higher IE than the source IR and VIS images, suggesting the fusion of information from sources to the VIS-IR composite images (FUS) and the two corrected images (COR1 and COR2). In terms of the MI shared with the original IR image, as indicated in Table 2, both corrected images maintain much higher mutual information with IR than the FUS (32.0% for COR1 and 28.6% for COR2). This demonstrates that both approaches incorporate more information from the source IR than the image-level fusion (FUS). The opposite trend is observed on comparing the MI with VIS among the three fused outcomes where COR1 and COR2 carry 9.16% and 6.67% less mutual information than FUS respectively. The higher MI with IR and lower MI with VIS reflect the fact that the physical correction process indeed favors the information from the IR image. Furthermore, comparing the two physical correction approaches, we find that COR2 has 2.63% less MI with IR and 2.74% more MI with VIS than COR1. This slight difference suggests that the additional first-step multiwavelet fusion associated with COR1 facilitates the incorporation of information from IR image into the final fused result.

Regarding to the change in radiation energy (AVGD and RMSD in Table 1), both COR1 and COR2 differ less from the original IR than the image-level fusion (FUS), supporting our objective to maintain the infrared thermal features using physical property-based correction. The radiation energy difference is greater for COR2 than COR1. We attributed this to the fact that COR1 has two steps to bring in more IR information compared with the direct correction for COR2.

The AG in Table 1 also indicates that the quality of the current approach (AG_COR2_ = 2.7804) lies between the previous correction method (AG_COR1_ = 2.9388) and image-level fusion (AG_FUS_ = 2.6771). The AG in all these cases is much higher than the original IR (AG_IR_ = 0.6205), suggesting that many sharp edges and details are added to the original IR to increase its spatial resolution. The additional image-processing step related to COR1 might explain the higher AG than COR2. By comparing the image similarity between COR1 and COR2 as indicated by QI and QE, we again find that COR2 shows greater similarity to VIS and less resemblance to IR than COR1.

Tables 3 and 4 present the results on 1300 LST 24 July 2006. A similar trend is observed. We also calculated the processing time of the current and previous methods. We used the data on 1300 LST 24 July 2006. The size of the VIS image was 3000 × 3000. The algorithm was written in MATLAB and was run on a PC with a 3.3-GHz Intel dual-core CPU and 2G RAM. The older method (Han2014) took 260.2 s to complete, during which 254.5 s were devoted to the first-step MRA image fusion. The current method took only 5.7 s. Obviously, by eliminating the time-consuming multiwavelet fusion, the current method was significantly more efficient.

In summary, these results show that the new method proposed here and Han2014 achieve very similar results, although Han2014 has slight superiority in several metrics, such as AVGD and RMSD. However, the new method is considerably more computationally efficient than Han2014, as no MRA processing is needed.

## 4. Conclusions

In this study, we propose a new algorithm that fuses the information of VIS images into IR images directly using the Stefan-Boltzmann Law as a constraint. Most existing fusion methods focus on visual effects, and many fail to incorporate the thermal physical properties of the IR images. The main objective of this study was to obtain higher-resolution IR images, while preserving the infrared thermal physical properties. The post-correction process uses the Stefan-Boltzmann Law as a strong constraint to ensure that the fused image has a radiation level similar to that of the original IR image. The VIS image is used directly as the input of this post-correction step.

In comparison with our previous method [15], which uses the multi-wavelet method to fuse VIS and IR images first followed by the same post-correction step using the Stefan-Boltzmann Law, this method yields an almost identical result, but it is considerably more efficient computationally, as no MRA processing is needed anymore. Therefore, this method brings a new perspective on the fusion of VIS and IR images.

This new algorithm is sensitive to the solar elevation angle. It performs best during the time period between 1100 and 1300 LST. At other times, the performance of this method will degrade due to the shadows in the visible channel image. It will be necessary to improve the method in the future by incorporating the influence of the angle between the sun and the satellite.

## Figures and Tables

**Table 1.** Quantitative analysis of IR and VIS image fusion and correction (1300 LST 24 July 2006). FUS, VIS-IR composite image based on multiwavelet fusion; COR1, corrected image based on VIS-IR composite, *i.e.*, FUS. COR1 is the final result of Han2014; COR2, corrected image based on the VIS image only.COR2 is the final result of the new method.

**Images**	**IE**	**AG**	**Q** *_abf_*	**AVGD**	**RMSD**
IR	6.9091	0.6502	-	-	-
VIS	4.9791	2.4797	-	-	-
FUS	7.0552	2.6771	0.6740	649.6730	906.2315
COR1	6.9934	2.9388	0.5407	366.8883	551.5277
COR2	6.9828	2.7804	0.5728	374.7601	562.7176

**Table 2.** Quantitative analysis of IR and VIS image fusion and correction (1300 LST 24 July 2006).

**Images**	**MI**	**QI**	**QE**
IR + FUS	1.3277	0.1829	0.0957
VIS + FUS	0.8830	0.8006	0.8114
IR + COR1	1.7530	0.1936	0.1045
VIS + COR1	0.8021	0.6969	0.6835
IR + COR2	1.7069	0.1445	0.0322
VIS + COR2	0.8241	0.7395	0.7307

**Table 3.** Quantitative analysis of IR and VIS image fusion and correction (1300 LST 25 July 2006).

**Images**	**IE**	**AG**	**Q** *_abf_*	**AVGD**	**RMSD**
IR	6.9567	0.6722	-	-	-
VIS	5.0491	2.1981	-	-	-
FUS	7.0574	2.4357	0.6451	578.3445	838.8759
COR1	7.0288	2.6651	0.5244	336.5270	516.8839
COR2	7.0168	2.4890	0.5540	346.6939	531.49323

**Table 4.** Quantitative analysis of IR and VIS image fusion and correction (1300 LST 25 July 2006).

**Images**	**MI**	**QI**	**QE**
IR + FUS	1.6075	0.2314	0.1405
VIS + FUS	0.9005	0.7447	0.7590
IR + COR1	2.0161	0.2382	0.1278
VIS + COR1	0.8724	0.6562	0.6478
IR + COR2	1.9398	0.1702	0.0395
VIS + COR2	0.8988	0.7112	0.7080
